# Clinical difference between fibroblast growth factor receptor 2 subclass, type IIIb and type IIIc, in gastric cancer

**DOI:** 10.1038/s41598-021-84107-x

**Published:** 2021-02-25

**Authors:** Masakazu Yashiro, Kenji Kuroda, Go Masuda, Tomohisa Okuno, Yuichiro Miki, Yurie Yamamoto, Tomohiro Sera, Atsushi Sugimoto, Shuhei Kushiyama, Sadaaki Nishimura, Shingo Togano, Masaichi Ohira

**Affiliations:** 1 Molecular Oncology and Therapeutics, Osaka City University Graduate School of Medicine, 1-4-3 Asahimachi, Abeno-ku, Osaka, 545-8585 Japan; 2 Department of Gastroenterological Surgery, Osaka City University Graduate School of Medicine, Osaka, Japan; 3 Cancer Center for Translational Research, Osaka City University Graduate School of Medicine, Osaka, Japan

**Keywords:** Tumour biomarkers, Tumour heterogeneity, Cancer, Gastrointestinal cancer, Gastric cancer

## Abstract

Fibroblast growth factor receptor 2 (FGFR2) has two isoforms: IIIb type and IIIc type. Clinicopathologic significance of these two FGFR2 subtypes in gastric cancer remains to be known. This study aimed to clarify the clinicopathologic difference of FGFR2IIIb and/or FGFR2IIIc overexpression. A total of 562 patients who underwent gastrectomy was enrolled. The expressions of FGFR2IIIb and FGFR2IIIc were retrospectively examined by immunohistochemistry or fluorescence in situ hybridization (FISH) using the 562 gastric tumors. We evaluated the correlation between clinicopathologic features and FGFR2IIIb overexpression and/or FGFR2IIIc overexpression in gastric cancer. FGFR2IIIb overexpression was observed in 28 cases (4.9%), and FGFR2IIIc overexpression was observed in four cases (0.7%). All four FGFR2IIIc cases were also positive for FGFR2IIIb, but not in the same cancer cells. FGFR2IIIb and/or FGFR2IIIc overexpression was significantly correlated with lymph node metastasis and clinical stage. Both FGFR2IIIb and FGFR2IIIc were significantly associated with poor overall survival. A multivariate analysis showed that FGFR2IIIc expression was significantly correlated with overall survival. FISH analysis indicated that *FGFR2* amplification was correlated with FGFR2IIIb and/or FGFR2IIIc overexpression. These findings suggested that gastric tumor overexpressed FGFR2IIIc and/or FGFR2IIIb at the frequency of 4.9%. FGFR2IIIc overexpression might be independent prognostic factor for patients with gastric cancer.

## Introduction

Fibroblast growth factor receptor 2 (FGFR2) is one of four FGFRs that encode a transmembrane receptor tyrosine kinase^1^. FGFR2 signaling is associated with the proliferation, migration, and angiogenesis of carcinomas^2,3^. FGFR2-overexpressed cancer might be a therapeutic target^4–6^. Several FGFR2 inhibitors have been developed for the treatment of cancer patients with enhanced expression of FGFR2 signaling^7–9^.

Gastric cancer is one of the most common malignancies in the world, and the prognosis of advanced gastric cancer remains to be poor^10^. The aberrant regulation of FGFR2 pathway has been implicated in anti-apoptosis, drug resistance, and epithelial-to-mesenchymal transition (EMT)^8^. *FGFR2* amplification has been reported in several types of solid carcinoma^11–13^ including gastric cancer^14–16^. We previously reported that the conditioned medium derived from gastric fibroblasts stimulates the growth of gastric cancer cells, which are mediated by FGF7/FGFR2 signaling^17,18^. Gastric cancers with *FGFR2* amplification, which was observed in 3–10% of all gastric cancers^19–24^, has been found to be associated with malignant progression^25–27^. Since FGFRs have an important role in the progression of gastric cancer, their use as a therapeutic candidate for the development of targeted anticancer agents should attract substantial attention^6,8,28^, while no clinical FGFR inhibitor has been approved. One of the reasons for the no clinical approvement of FGFR inhibitors for gastric cancer treatment might be a lack of tool that allows optimal patient selection. The establishment of beneficial markers is necessary to select for FGFR-targeted therapy^2,29^.

To date, the molecular heterogeneity has been gradually elucidated^30,31^. Following the understanding of gastric cancer biology, targeted therapies have been evaluated in experimental studies and transferred promptly to clinical trials. FGFR2 has two isoforms, i.e., the IIIb type and the IIIc type based on the alternative splicing within the C-terminal half of the third Ig loop (D3) in the extracellular FGF binding domain, which are alternatively spliced by exon 8 and by exon 9, respectively^1,7,32^. The spliced isoforms differ in binding ligands: FGFR2IIIb is a high-affinity receptor for FGF1, -3, -7, -10, and -22, whereas FGFR2IIIc binds FGF1, -2, -4, -6, -8, -9 -17 and -18^33,34^. The FGFR2IIIb isoform is expressed mainly in epithelial cells, and it preferentially binds secreted FGF ligands from adjacent mesenchymal cells^33^. In contrast, the FGFR2IIIc isoform is preferentially expressed in mesenchymal cells and usually binds ligands secreted from the adjacent epithelial cells^35^. These findings suggested that the clinical significance of FGFR2 signaling in cancer might differ between FGFR2IIIb and FGFR2IIIc.

There are a few reports of FGFR2IIIb and FGFR2IIIc expression in some other types of solid cancers including gastric carcinomas^36–38^. In this study, we conducted to examine the clinicopathologic significance of the expression of FGFR2IIIb and that of FGFR2IIIc using a large sample of gastric cancer.

## Results

### FGFR2IIIb-positive and FGFR2IIIc-positive patients

**Figure 1:**
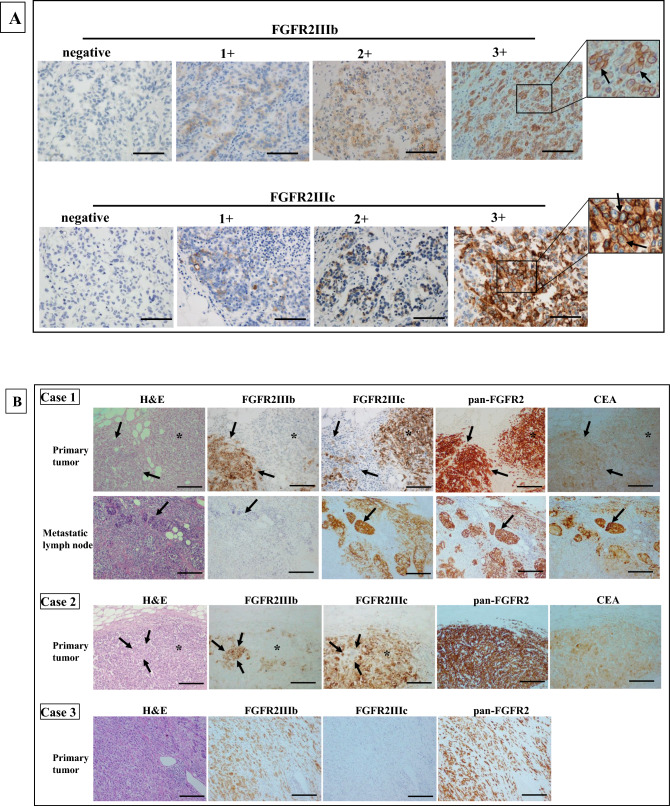
Representative pictures of FGFR2IIIb and FGFR2IIIc expression in gastric cancer. (**A**) IHC score of FGFR2IIIb and FGFR2IIIC. FGFR2IIIb and FGFR2IIIc were mainly expressed at cell membrane (arrows and inset). The immunoreactivity of FGFR2IIIb and FGFR2IIIc were evaluated according to the intensity of membranous staining score 0–3, as follows: 0, negative; 1 +, weak at cytosol; 2 +, moderate at cell membrane; 3 +, strong at cell membrane. FGFR2IIIb and FGFR2IIIc expression were considered positive when scores were ≥ 2. Bar = 100 μm. (**B) **Representative cases stained with H&E, FGFR2IIIb, FGFR2IIIc, pan-FGFR2, and CEA of gastric cancer. Heterogeneous expressions of FGFR2IIIb isoform and FGFR2IIIc isoform were found in a tumor (Case 1 and Case 2). The pan-FGFR2 detected both FGFR2IIIb isoforms and FGFR2IIIc isoforms. FGFR2IIIb expression (arrows) and FGFR2IIIc expression (asterisks) were positive in the same tumor, but both were not positive in the same tumor cells. FGFR2IIIc dominant expression in a metastatic lymph node disease was found in Case 1. CEA determined cancer cells in the primary tumor and the lymph node. In contrast, Case 3 shows FGFR2IIIb expression, but not FGFR2IIIc expression. × 100. Bar = 200 μm.

FGFR2IIIb and FGFR2IIIc were mainly expressed at the cell membrane of the cancer cells (Fig. 1A). Among the 562 gastric cancers, FGFR2IIIb and FGFR2IIIc were positive in 28 cases (4.9%) and four cases (0.7%), respectively. All four FGFR2IIIc-positive tumors were also positive for FGFR2IIIb in the same tumor, but both were not positive at the same cancer cells. Case 1 and Case 2 showed heterogeneous expression of both FGFR2IIIb isoform and FGFR2IIIc isoform in a primary tumor (Fig. 1B). In contrast, most of FGFR2-positive tumors were positive for FGFR2IIIb but not FGFR2IIIc (Case 3). The relationships between the clinicopathologic features and the FGFR2IIIb expression or FGFR2IIIc expression were summarized in Table 1. There was a significant correlation between FGFR2IIIb expression and Borrmann's type 4 (p < 0.001), undifferentiated type, depth of invasion (p < 0.001), lymph node metastasis (p < 0.001), lymphatic invasion (p < 0.001), venous invasion (p = 0.015), and clinical stage (p < 0.001). FGFR2IIIc expression was significantly correlated with lymph node metastasis (p = 0.039) and clinical stage (p = 0.014).

**Table 1:** Association between FGFR2-IIIb or FGFR2-IIIc expression and clinicopathologic factors in 562 primary gastric carcinomas.

Clinicopathologic features	FGFR2-IIIb type			FGFR2-IIIc type		
	Positive	Negative	p-value	Positive	Negative	p-value
	(n = 28)	(n = 534)		(n = 4)	(n = 558)	
**Age**						
< 60	8 (4.8%)	159 (95.2%)	0.892	0 (0%)	167 (100%)	0.244
≥ 60	20 (5.1%)	375 (94.9%)		4 (1.0%)	391 (99.0%)	
**Gender**						
Female	10 (4.1%)	233 (95.9%)	0.41	2 (0.8%)	241 (99.2%)	0.580
Male	18 (5.6%)	301 (94.4%)		2 (0.6%)	317 (99.4%)	
**Macroscopic type**						
Borrmann’s type 4	10(17.5%)	47 (82.5%)	< 0.001	2 (3.5%)	55 (96.5%)	0.052
Other types	18 (3.6%)	487 (96.4%)		2 (0.4%)	503 (99.4%)	
**Microscopic type**						
Differentiated	4 (1.5%)	268 (98.5%)	< 0.001	0 (0%)	272 (100%)	0.069
Undiffrentiated	24 (8.3%)	266 (91.7%)		4 (1.4%)	286 (98.6%)	
**T stage**						
T1	1 (0.4%)	254 (99.6%)	< 0.001	0 (0%)	255 (100%)	0.087
T2,3,4	27 (8.8%)	280 (91.2%)		4 (1.3%)	303 (98.7%)	
**Lymph node metastasis**						
Negative	4 (1.3%)	308 (98.7%)	< 0.001	0 (0%)	312 (100%)	0.039
Positive	24 (9.6%)	226 (90.4%)		4 (1.6%)	246 (98.4%)	
**Hepatic metastasis**						
Negative	26 (4.7%)	523 (95.3%)	0.081	3 (0.5%)	546 (99.5%)	0.090
Positive	2 (15.4%)	11 (84.6%)		1 (7.7%)	12 (92.3%)	
**Peritoneal dissemination**						
Negative	26 (4.9%)	503 (95.1%)	0.769	4 (0.8%)	525 (99.2%)	0.784
Positive	2 (6.1%)	31 (93.9%)		0 (0%)	33 (100%)	
**Metastasis**						
Negative	27 (4.9%)	520 (95.1%)	0.761	4 (0.7%)	543 (99.3%)	0.897
Positive	1 (6.7%)	14 (93.3%)		0 (0%)	15 (100%)	
**Lymphatic invasion**						
Negative	1 (0.4%)	241 (99.6%)	< 0.001	0 (0%)	242 (100%)	0.104
Positive	27 (8.4%)	293 (91.6%)		4 (1.3%)	316 (98.7%)	
**Venous invasion**						
Negative	18 (3.9%)	441 (96.1%)	0.015	2 (0.4%)	457 (99.6%)	0.1
Positive	10 (9.7%)	93 (90.3%)		2 (1.9%)	101 (98.1%)	
**Clinical stage**						
I & II	5 (1.4%)	362 (98.6%)	< 0.001	0 (0%)	367 (100%)	0.014
III & IV	23 (11.8%)	172 (88.2%)		4 (2.1%)	191 (97.9%)	

### FGFR2IIIb and FGFR2IIIc expression on lymph nodes

Among the 28 gastric cancers with FGFR2IIIb and/or FGFR2IIIc expression, 25 cases had lymph node metastasis. FGFR2IIIb expression on lymph nodes was observed in 10 (40%) of the 25 cases with lymph node metastasis. FGFR2IIIc expression on lymph nodes was observed in 4 (16%) of the 25 cases, and the 4 cases were the same cases with FGFR2IIIc expression at the primary tumor. All four cases with FGFR2IIIc expression were also positive for FGFR2IIIb expression in lymph nodes, but both were not positive at the same cancer cells, which was as well as the primary tumors. In Case 1, FGFR2IIIc dominant expression was found in a metastatic lymph node (Fig. 1B).

### FGFR2 gene amplification analyzed by FISH

**Figure 2:**
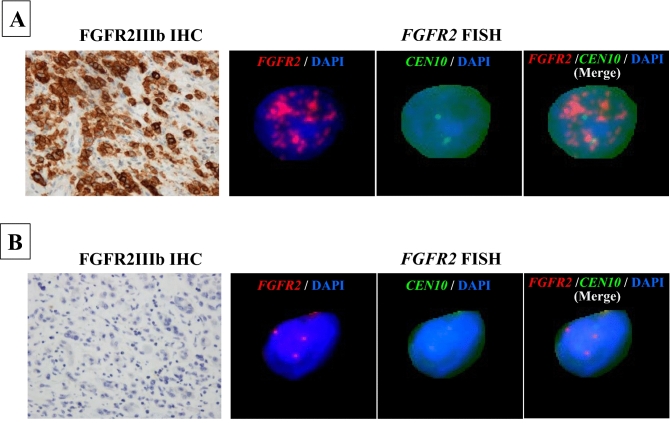
*FGFR2* amplification by FISH. (**A**) This sample designated FGFR2IIIb IHC score 3 + showed *FGFR2* amplification (*FGFR2/CEN10* ratio = 11.79). (**B)** This sample designated FGFR2IIIb IHC score 0 didn’t show *FGFR2* amplification (*FGFR2/CEN10* ratio = 1.06).

A total of 33 gastric cancer including 28 FGFR2-positive tumors and five FGFR2-negative tumors were examined *FGFR2* amplification by FISH. Figure 2 Eleven (39.3%) of the 28 FGFR2-IIIb positive cases had *FGFR2* gene amplification: *FGFR2* amplification was observed in nine of the 11 cases with an immunohistochemistry score of 3 +, two of the 17 cases with a score of 2 +, and none of the five cases with a score of 1 + or 0. *FGFR2/CEN10* ratio of cancer cells with FGFR2IIIb and/or FGFR2IIIc overexpression was significantly (p < 0.01) correlated to that of IHC score by immunohistochemical staining (Table 2). All four cases with FGFR2IIIc expression had *FGFR2* amplification. The average FGFR2/CEN10 ratio was 9.1 ± 4.8 in the 11 cases of immunohistochemistry score 3 + , 1.8 ± 2.3 in the 17 cases of score 2 +, and 1.0 ± 0.081 in the five cases with score 1 + and/or 0.

**Table 2:** Correlation between *FGFR2/CEN10* ratio and intensity of immunohistochemical staining.

*FGFR2/CEN10* ratio	IHC score			p value
	3 +	2 +	1 +/0	
≥ 2.0	9	2	0	
< 2.0	2	15	5	< 0.001

### Correlation between FGFR2 expression/amplification and the patients' survival

**Figure 3:**
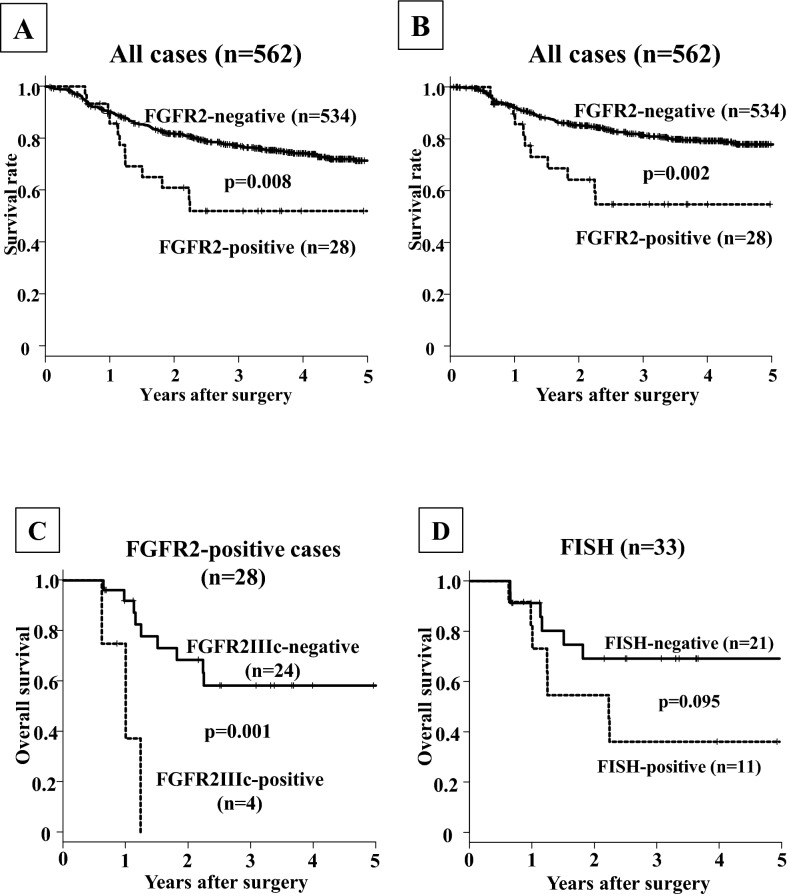
Survival curve. (**A**) Overall survival of patients with FGFR2IIIb-positive tumor was significantly poorer (p = 0.008) than that of patients with FGFR2IIIb-negative tumor. (**B**) Disease specific survival of patients with FGFR2IIIb-positive tumor was significantly poorer (p = 0.002) than that of patients with FGFR2IIIb-negative tumor. (**C**) In patients with FGFR2-positive tumor, overall survival of patients with FGFR2IIIc-positive tumor was significantly (p = 0.003) poorer than that with FGFR2IIIc-negative. (**D**) *FGFR2* amplification tended to be associated with a poorer outcome, compared with non-amplified cases (p = 0.095).

The overall survival and disease-specific survival of the gastric cancer patients with FGFR2IIIb and/or FGFR2IIIc expression (n = 28) was significantly poorer than that of the patients with FGFR2-negative expression (n = 534) (p = 0.008 and 0.002, respectively) (Fig. 3A,B). Among the 28 patients with an FGFR2-positive tumor, the survival rate of the patients who were positive for FGFR3IIIc (n = 4) was significantly poorer than that of FGFR2IIIc-negative patients (n = 24) (p = 0.003, log-rank; Fig. 3C). *FGFR2* amplification cases (n = 11) tended to be associated with a poorer outcome (log-rank test, p = 0.095) compared to the non-amplified cases (n = 22) (Fig. 3D).

The univariate analysis revealed that the overall survival was significantly correlated with FGFR2IIIb expression, FGFRIIIc expression, invasion depth, macroscopic type, histological type, lymph node metastasis, peritoneal dissemination, lymphatic invasion, and cytology. Among these clinicopathologic factors, the multivariate analysis revealed that FGFR2IIIc expression (p = 0.045), macroscopic type (p < 0.001), invasion depth (p = 0.003), lymph node metastasis (p < 0.001), hepatic metastasis (p = 0.005), and peritoneal dissemination (p < 0.001) was significantly correlated with overall survival (Table 3).

**Table 3:** Univariate and multivariate analysis with respect to overall survival.

Parameter	Univariate analysis			Multivariate analysis		
	Hazard ratio	95% CI	p-value	Hazard ratio	95% CI	p-value
**FGFR2-IIIb expression**						
Positive versus negative	2.17	1.20–3.93	0.010	0.65	0.32–1.31	0.226
**FGFR2-IIIc expression**						
Positive versus negative	7.64	2.40–24.3	< 0.001	3.92	1.02–15.2	0.045
**Age**						
≥ 60 versus < 60	1.50	1.04–2.17	0.032	1.43	0.97–2.10	0.067
**Gender**						
Female versus male	1.20	0.86–1.67	0.276			
**Macroscopic type**						
Type 4 versus Other types	7.18	5.01–10.3	< 0.001	2.53	1.67–3.83	< 0.001
**Microscopic type**						
Differentiated versus undiffrentiated	1.71	1.23–2.37	0.001	1.07	0.75–1.54	0.697
**Depth of invasion**						
T1 versus T2,3,4	7.59	4.69–12.3	< 0.001	2.45	1.35–4.46	0.003
**Lymph node metastasis**						
Negative versus Positive	8.27	5.42–12.6	< 0.001	3.33	1.97–5.62	< 0.001
**Hepatic metastasis**						
Negative versus Positive	5.50	2.89–10.5	< 0.001	2.60	1.33–5.08	0.005
**Peritoneal dissemination**						
Negative versus Positive	8.17	5.26–12.7	< 0.001	2.81	1.74–4.56	< 0.001
**Lymphatic invasion**						
Negative versus Positive	5.44	3.49–8.49	< 0.001	1.12	0.64–1.95	0.693
**Venous invasion**						
Negative versus Positive	3.14	2.25–4.38	< 0.001	1.20	0.83–1.72	0.332

## Discussion

FGFR2 has two isoforms, IIIb type and the IIIc type, which are alternatively spliced by exon 8 and by exon 9, respectively^39^. In this study, we used two new mouse-rat chimeric monoclonal antibodies, mFR2-10b and mFR2-28c, which recognize human cells with FGFR2IIIb overexpression and FGFR2IIIc overexpression, respectively. Also, we compared the IHC results for both FGFR2IIIb and FGFR2IIIc isoforms using an available pan-FGFR2 antibody, which supported specificity of protein isoform expression. Both antibodies stained the cell membrane of the cancer cells with *FGFR2* amplification. These findings suggested that the two new recombinant chimeric antibodies, mFR2-10b against FGFR2IIIb and mFR2-28c against FGFR2IIIc, might be useful for the determination of carcinomas with FGFR2IIIb isoform and FGFR2IIIc isoform. The scoring system of FGFR2IIIb and FGFR2IIIc immunoreactivity were evaluated according to the intensity of membranous staining in the deepest level of the tumor cells. Therefore, scoring system will allow reporting possible heterogeneity of FGFR IIIb and IIIc in a case, but not in the individual tumor cells.

*FGFR2*, one of the known driver genes of solid tumors, has been a therapeutic target for gastric cancer^9,40,41^. We also have reported that an FGFR2 phosphorylation inhibitor is useful for the treatment of gastric cancer with *FGFR2* amplification^18,41,42^. Although a reliable biomarker is desired to identify the gastric cancer patients who are candidates for FGFR2 targeting therapy, however suitable FGFR2 antibodies that are specific for the FGFR2IIIb and FGFR2IIIc types are not yet available, so far. We herein obtained that FGFR2IIIb and FGFR2IIIc antibodies specifically bind to the cell membrane of cancer cells. In human gastric carcinomas, FISH analysis results also indicated that *FGFR2* amplification was correlated with FGFR2IIIb overexpression and/or FGFR2IIIc overexpression. In this study, our two antibodies, FGFR2IIIb and FGFR2IIIc, might be useful tool for the determination of carcinomas with FGFR2 overexpression and treatment of FGFR2 inhibitors. Analyses of FGFR2 expression using these antibodies may be reliable for the identification of patients who are appropriate for FGFR2 targeting therapy.

Our present analyses demonstrated that the overexpression of FGFR2IIIb and/or FGFR2IIIc was 4.9% in 562 gastric cancers. In human gastric carcinomas, FISH analysis results also indicated that *FGFR2* amplification was correlated with FGFR2IIIb overexpression and/or FGFR2IIIc overexpression. Several immunohistochemical studies previously reported that FGFR2 overexpression ranged from 10 to 51% of gastric cancer cases^43–46^. *FGFR2* amplification has been reported to be observed in 3%–10% of gastric cancers^20–24^. Currently, several studies have defined the genomic landscape of GC using the next-generation sequencing (NGS) technology^47–51^, and indicated that the genetic alternations of *FGFR2* is around 3–10%^50^. These findings might suggest that FGFR2 overexpression might be present in around 5% of all gastric cancers, which is lower than previous reports.

Since FGFR2 has two isoforms, IIIb type and IIIc type, it is important to examine the frequency of FGFR2-IIIc expression among FGFR2 overexpressing gastric cancers. However few reports of FGFR2 subclass of IIIb and IIIc expression are available in gastric cancer^36^. In this study, the overexpression of FGFR2IIIb was 4.9% and that of FGFR2IIIc was 0.7% in 562 gastric cancers. FGFR2IIIb-positive expression was frequently observed in the cases with Borrmann's type 4, undifferentiated type, high invasion depth, lymph node metastasis, lymphatic invasion, venous invasion, and clinical stage. The prognosis of the FGFR2IIb-positive patients was significantly poorer compared to that of the patients with FGFR2IIIb-negative cancer. These findings may indicate that FGFR2IIIb signaling is associated with the malignant progress of gastric cancer. In contrast, no report of FGFR2IIIc expression in gastric cancer is available, whereas It has been reported that FGFR2IIIc immunoreactivity was expressed in 49% and 27% in endometrial endometrioid carcinoma^52^ and colorectal carcinoma^12,37^. The expression rate of FGFR2IIIc in 562 gastric cancer cases was only 0.7%, suggesting that gastric cancer expressed FGFR2IIIc extremely few in compared to the expression rate of FGFR2IIIb. These findings suggested that FGFR2IIIb signaling inhibition might be more wide indication for GC patients bearing FGFR2.

Several FGFR2 tyrosine kinase inhibitors (TKIs), including AZD4547 (NCT01457846, SHINE trial), dovitinib (NCT01719549), E7090 (NCT02275910), and TAS-120 (NCT02052778), have been evaluated for patients of gastric cancer with *FGFR2* amplification^16,40,41,53^. Recently, FGFR2 specific antibodies, FPA144 (NCT02318329) have been developed to treat patients bearing high FGFR2b-overexpressing gastric cancers. FPA144, Bemarituzumab, is glycol-engineered for enhanced antibody-dependent cell-mediated cytotoxicity (ADCC), is currently being evaluated in a phase III trial as front-line therapy for patients with FGFR2-IIIb overexpressing advanced-stage^54,55^. FGFR2 signaling is associated with the proliferation, migration, and angiogenesis of carcinomas^11,17^, therefore, these FGFR2 signaling inhibitors have been proposed to be a key drug for the treatment of solid tumors with FGFR2 overexpression. However several studies of FGFR2 inhibitors did not improve the prognosis of GC patients. *FGFR2* gene amplification was detected in 39.3% of our FGFR2IIIb-positive cases and all of the FGFR2IIIc-positive cases. All FGFR2IIIc-positive tumors were also positive for FGFR2IIIb in the same tumor, but both were not positive in the same cancer cells. One of the reasons for the negative results might be this tumor heterogeneity. Kim et al.^56^ reported that a difference in sensitivity to FGFR2 inhibitor of AZD4547 may be related to baseline FGFR2 IIIC expression level caused by tumor heterogeneity using patient-derived tumor cell models. The molecular heterogeneity of FGFR2 IIIb type and FGFR2 IIIc might be one of the mechanisms of resistance of FGFR2 signaling therapy.

Among our 28 patients with FGFR2IIIb-positive gastric cancer, the prognoses of the four FGFR2IIIc-positive patients were significantly poorer than those of the 24 FGFR2IIIc-negative patients, and FGFR2IIIc was an independent prognostic factor in this study. FGFR2IIIc expression was reported to be correlated with the EMT^32,57^. In our present cohort, FGFR2IIIc expression was frequently observed in the cases with lymph node disease compared to the expressions in the primary tumors. These findings indicated that FGFR2IIIc signaling might be associated with metastatic ability of gastric cancer cells. EMT is a critical process in cancer progression that provides cancer cells with the ability to escape from the primary foci, invade stromal tissues, and metastasize to secondary regions due to decreased cell–cell adhesion^58,59^.

All FGFR2IIIc-positive tumors were also positive for FGFR2IIIb in the same tumor, but both were not positive in the same cancer cells. The IIIb type and the IIIc type are alternatively spliced by exon 8 and by exon 9, respectively^39^. We previously reported that splicing variant of FGFR2 IIIb type and FGFR2 IIIc type might be regulated the oxidative level of tumor microenvironment^25^. Although both FGFR2IIIc expression and FGFR2IIIb expression were observed in the same tumors, they were not positive in the same cells. In normal human tissues, FGFR2IIIb is expressed mainly on epithelial cells whereas FGFR2IIIc is expressed mostly in mesenchymal cells^35^. FGFR2IIIc signaling might be associated with the EMT, which is one of the malignant phenotypes of cancer progression. FGFR2IIIc expression was frequently observed in lymph node disease in the present study, and it was significantly correlated with poor survival. These findings might indicate that the switch from FGFR2IIIb to FGFR2IIIc is one of the mechanisms underlying the malignant progression of gastric cancer cells.

In conclusion, FGFR2IIIc and/or FGFR2IIIb overexpression was observed in 4.9% of gastric cancer. FGFR2IIIc might be useful independent prognostic marker for patients with gastric cancer.

## Methods

### Clinical materials

A total of 562 patients who were histologically confirmed to have primary gastric cancer was enrolled in this study. All patients underwent a resection of gastric tumor and regional lymph nodes at Osaka City University. None of patients had undergone preoperative radiation and/or chemotherapy. The pathologic diagnoses and classifications were made according to the UICC TNM classification of malignant tumors. This study was approved by Osaka City University ethics committee (Reference number 924). Informed consent was obtained from all patients. All methods with respect to humans were carried out in accordance with relevant guidelines and regulations.

### Antibodies

Mouse-rat chimeric monoclonal antibodies which recognize human FGFR2-IIIb (mFR2-10b) and human FGFR2-IIIc (mFR2-28c) were produced and provided from Daiichi Sankyo Co., Ltd. as follows. Female WKY/Izm rats (Japan SLC, Inc.) were immunized with recombinant human FGFR2b (IIIb)/Fc Chimera or FGFR2c (IIIc)/Fc Chimera (R&D Systems, Inc.), and the lymphoid cells or splenocytes collected from immunized rats were fused with SP2/0-Ag14 mouse myeloma cells (ATCC: CRL-1581) by conventional hybridoma techniques. Hybridoma culture supernatants obtained from the established hybridomas were screened for FGFR2 binding by the flowcytometry analysis using FGFR2-expressing cells. The selected established hybridomas were further screened for their ability to apply immunohistochemistry using FGFR2-expressing cells embedded in FFPE. Rat FR2-10b (FGFR2IIIb specific) and rat FR2-28c (FGFR2IIIc specific) were selected (Supplement Fig. S1), and their variable regions were cloned and fused into expression vector containing constant region of mouse IgG1. The recombinant chimeric antibodies of mouse FR2-10b (mFR2-10b) and mouse FR2-28c (mFR2-28c) were produced in culture supernatant from FREESTYLE 293F cells (Thermo Fisher Scientific Inc.), and purified by MABSELECTSURE column chromatography (GE Healthcare Bioscience). All methods with respect to animals were carried out in accordance with relevant guidelines and regulations. An Anti-CEA antibody (sc-48364; 1:200, Santa Cruz, Dallas, TX, USA) was used to determine histologically cancer cells in the tumor tissue. A pan-FGFR2 antibody (#23328, 1:200; Cell Signaling, Danvers, MA, USA) was used to detect cancer cells with both FGFR2IIIb and/or FGFR2IIIc expression. All methods with respect to animals were carried out in accordance with relevant ARRIVE guidelines.

### Immunostaining of the FGFR2-IIIb and FGFR2-IIIc

Immunohistochemical study was performed as follows. In brief, Paraffin-embedded sections from 562 patients were heated for 40 min at 98 °C by heater in Target Retrieval Solution High pH (DAKO, Carpinteria, CA). Then sections were incubated with 3% hydrogen peroxide to block endogenous peroxidase activity and the sections were immersed in protein block (DAKO protein block, serum free) to block nonspecific binding. The specimens were incubated with anti-FGFR2-IIIb (dilution, 1:333) for 30 min at room temperature, and anti-FGFR2-IIIc (dilution, 1:333) for overnight at 4 °C. These sections were incubated with mouse linker (ENVISION FLEX Mouse+, DAKO) for 20 min, and peroxidase-labeled polymer (ENVISION FLEX/HRP, DAKO) for 20 min. The sections were counterstained with hematoxylin.

### Immunohistochemical determination

The immunoreactivity of FGFR2IIIb and FGFR2IIIc were evaluated according to the intensity of membranous staining in the deepest level of the tumor. FGFR2IIIb and FGFR2IIIc were mainly expressed at the cell membrane and weakly expressed in the cytosol of cancer cells. Immunostaining intensity score was rated 0–3 as follows: 0, negative; 1 +, weak at cytosol; 2 +, moderate at cell membrane; 3 +, strong at cell membrane (Fig. 1A). FGFR2IIIb or FGFR2IIIc expression were considered positive when scores were ≥ 2.

### Fluorescence in situ hybridization (FISH) of FGFR2

All of 28 FGFR2-positive tumors and 5 of FGFR2-negative tumors were examined *FGFR2* gene amplification by FISH analysis. The area most strongly immunostained of tumor paraffin-embedded sections were subjected to FISH. Tumor sections were cut to 4 μm thickness, followed by deparaffinization with the pretreatment reagent (Abbott Molecular Inc., 2J06-30) at 80 ± 1 °C for 30 min. Protease digestion procedures were performed using the protease reagent (Abbott Molecular Inc., 2J08-32) at 37 ± 1 °C for 60 min. FGFR2/CEN10p Dual Color FISH Probe conjugated with Texas Red/FITC (GSPLab., Inc, GC018) were hybridized at 75 °C for 5 min, and 37 °C for 48 h (Supplement Fig. S2). After hybridization, the slides were washed in 2 × saline-sodium citrate/0.3% NP-40 at 72 °C for 5 min, and counterstained with PROLONG Gold antifade reagent with DAPI (Thermo Fischer Scientific, P36935). The specimens were examined with ARIOL SL-200 (Leica Biosystems). FGFR2/CEN10 signals of 40 tumor cell nuclei were counted and FGFR2/CEN10 ratio was calculated according to the evaluation method of The PATHVYSION HER-2 DNA Probe Kit (Abbott). An FGFR2/CEN10 ratio 2.0 or higher were defined as gene amplification positive.

### Statistical analysis

We used the χ^2^ test or Fisher’s exact test to determine the significance of the difference between the covariates. The survival durations were calculated using the Kaplan–Meier method and analyzed by the log-rank test to compare the cumulative survival durations in the patient groups. In addition, the Cox proportional hazards model was used to compute multivariate hazards ratios for the study parameters. In all of the tests, a *p* value < 0.05 was defined as statistically significant. Statistical analysis was conducted in R (R Project for Statistical Computing, V.3.5.3).

## Supplementary Information

Supplementary Information 1.

Supplementary Information 2.
