# Supplementary material for: Clinical difference between fibroblast growth factor receptor 2 subclass, type IIIb and type IIIc, in gastric cancer
Source: Sci Rep. 2021 Feb 25;11:4698. doi: 10.1038/s41598-021-84107-x (PMC7907198; doi:10.1038/s41598-021-84107-x)
Supplement: Supplementary file 1 — Supplementary Information 1. [file 41598_2021_84107_MOESM1_ESM.pdf]

Clinical Difference Between Fibroblast Growth Factor Receptor 2 Subclass, Type IIIb and Type IIIc, in Gastric Cancer

Masakazu Yashiro[1,2,3], Kenji Kuroda[1,2], Go Masuda[1,2], Tomohisa Okuno[1,2], Yuichiro Miki[1,2], Yurie Yamamoto[1], Tomohiro Sera[1,2], Atsushi Sugimoto[1,2], Shuhei Kushiyama[1,2], Sadaaki Nishimura[1,2], Shingo Togano[1,2], and Masaichi Ohira[2]

[1] Molecular Oncology and Therapeutics, Osaka City University Graduate School of Medicine. [2] Department of Gastroenterological Surgery, Osaka City University Graduate School of Medicine. [3] Cancer Center for Translational Research, Osaka City University Graduate School of Medicine, Osaka, Japan

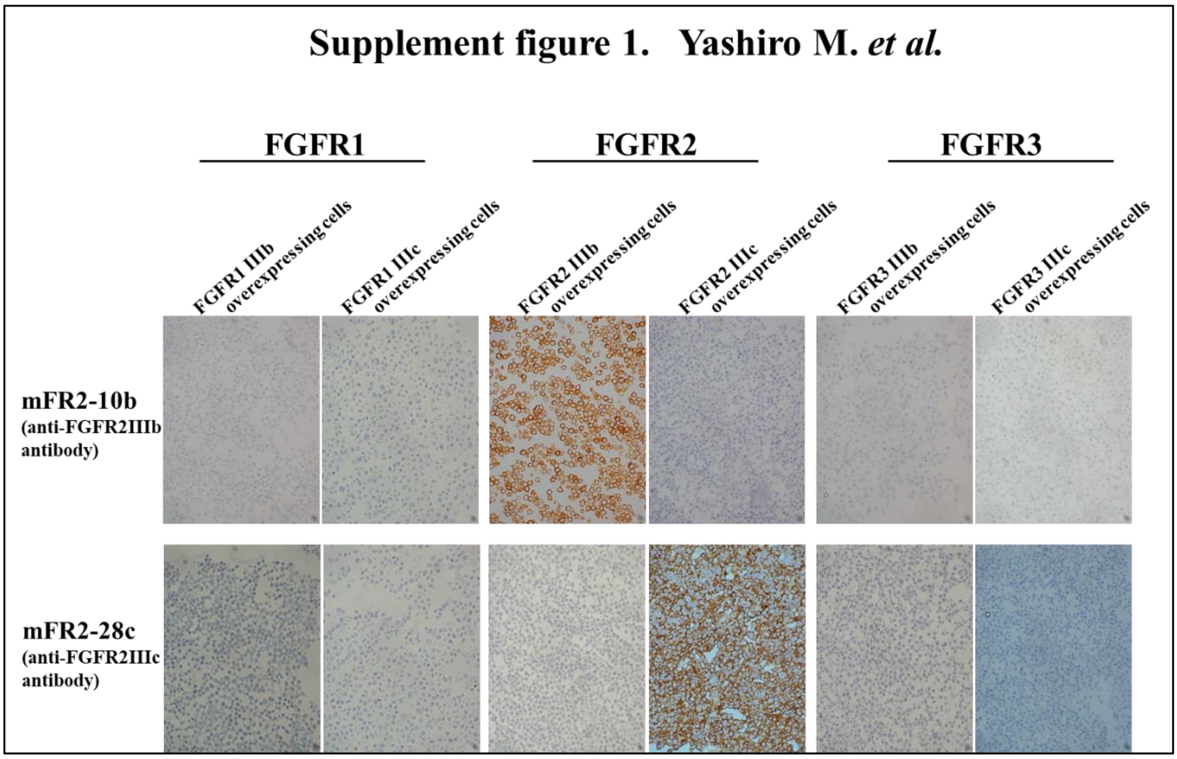

Supplement figure 1. Representative pictures of staining by mFR2-10b and mFR2-28c on FGFR2IIIb and FGFR2IIIc overexpression cells. The recombinant chimeric antibodies of mFR2-10b and mouse mFR2-28c recognized FGFR2IIIb and FGFR2III overexpression cells, respectively, but not other FGFR overexpression cells.
