# Supplementary material for: Clinical difference between fibroblast growth factor receptor 2 subclass, type IIIb and type IIIc, in gastric cancer
Source: Sci Rep. 2021 Feb 25;11:4698. doi: 10.1038/s41598-021-84107-x (PMC7907198; doi:10.1038/s41598-021-84107-x)
Supplement: Supplementary file 2 — Supplementary Information 2. [file 41598_2021_84107_MOESM2_ESM.pdf]

Clinical Difference Between Fibroblast Growth Factor Receptor 2 Subclass, Type IIIb and Type IIIc, in Gastric Cancer

Masakazu Yashiro[1,2,3], Kenji Kuroda[1,2], Go Masuda[1,2], Tomohisa Okuno[1,2], Yuichiro Miki[1,2], Yurie Yamamoto[1], Tomohiro Sera[1,2], Atsushi Sugimoto[1,2], Shuhei Kushiyama[1,2], Sadaaki Nishimura[1,2], Shingo Togano[1,2], and Masaichi Ohira[2]

[1] Molecular Oncology and Therapeutics, Osaka City University Graduate School of Medicine. [2] Department of Gastroenterological Surgery, Osaka City University Graduate School of Medicine. [3] Cancer Center for Translational Research, Osaka City University Graduate School of Medicine, Osaka, Japan

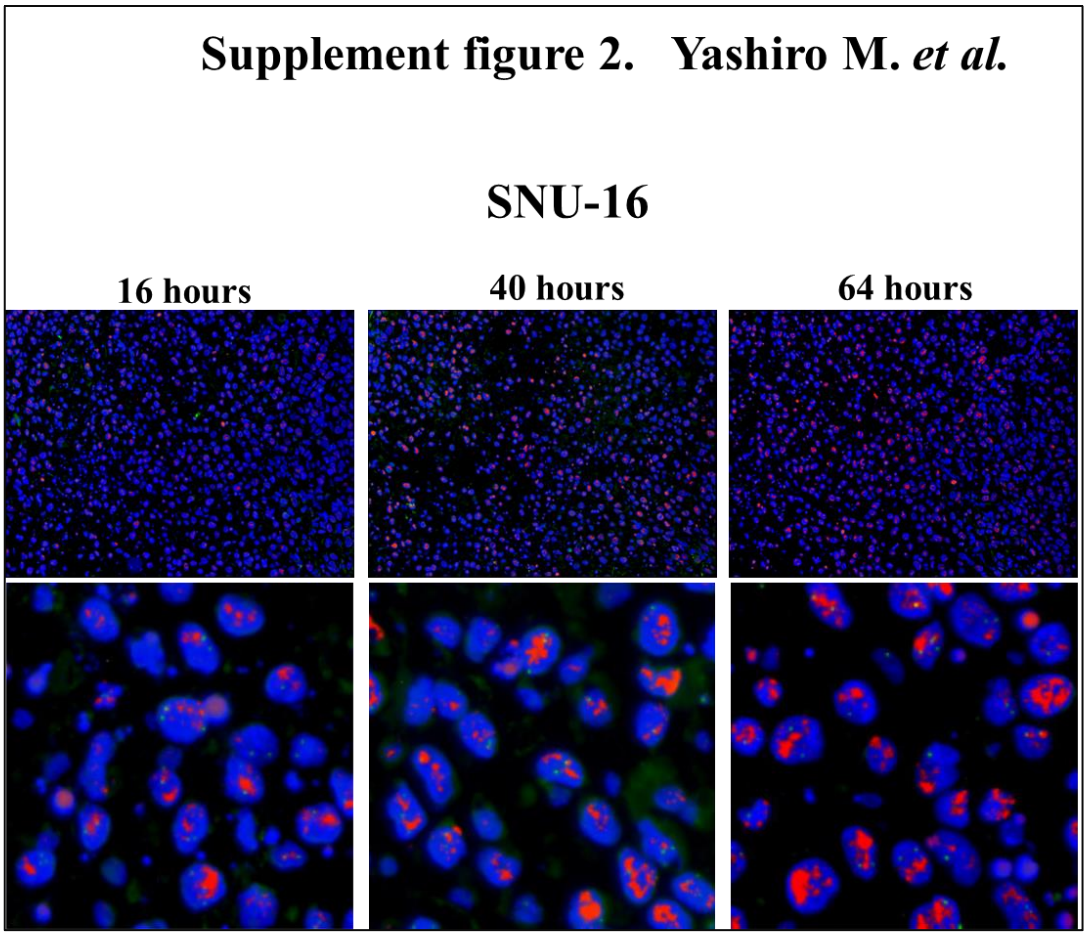

Supplement figure 2. FISH analysis by hybridization of FGFR2 and CEN10p. Probe FGFR2/CEN10p Dual Probe conjugated with Texas Red/FITC were hybridized on SNU-16 cells for 16, 40, and 64 hours. Hybridization time for 40 hours was determined to be adequate.
